# The perception of caricatured emotion in voice

**DOI:** 10.1016/j.cognition.2020.104249

**Published:** 2020-07

**Authors:** Caroline M. Whiting, Sonja A. Kotz, Joachim Gross, Bruno L. Giordano, Pascal Belin

**Affiliations:** a Institute of Neuroscience and Psychology, University of Glasgow, Glasgow, UK; b Faculty of Psychology and Neuroscience, Department of Neuropsychology and Psychopharmacology, Maastricht University, Maastricht, the Netherlands; c Department of Neuropsychology, Max Planck Institute for Human Cognitive and Brain Sciences, Leipzig, Germany; d Institute for Biomagnetism and Biosignalanalysis, University of Münster, Germany; e Institut de Neurosciences de la Timone, CNRS UMR 7289, Aix-Marseille Université, Marseille, France

**Keywords:** Nonverbal vocalisations, Vocal affect, Caricatured emotions, Emotion perception

## Abstract

Affective vocalisations such as screams and laughs can convey strong emotional content without verbal information. Previous research using morphed vocalisations (e.g. 25% fear/75% anger) has revealed categorical perception of emotion in voices, showing sudden shifts at emotion category boundaries. However, it is currently unknown how further modulation of vocalisations beyond the veridical emotion (e.g. 125% fear) affects perception. Caricatured facial expressions produce emotions that are perceived as more intense and distinctive, with faster recognition relative to the original and anti-caricatured (e.g. 75% fear) emotions, but a similar effect using vocal caricatures has not been previously examined. Furthermore, caricatures can play a key role in assessing how distinctiveness is identified, in particular by evaluating accounts of emotion perception with reference to prototypes (distance from the central stimulus) and exemplars (density of the stimulus space). Stimuli consisted of four emotions (anger, disgust, fear, and pleasure) morphed at 25% intervals between a neutral expression and each emotion from 25% to 125%, and between each pair of emotions. Emotion perception was assessed using emotion intensity ratings, valence and arousal ratings, speeded categorisation and paired similarity ratings. We report two key findings: 1) across tasks, there was a strongly linear effect of caricaturing, with caricatured emotions (125%) perceived as higher in emotion intensity and arousal, and recognised faster compared to the original emotion (100%) and anti-caricatures (25%–75%); 2) our results reveal evidence for a unique contribution of a prototype-based account in emotion recognition. We show for the first time that vocal caricature effects are comparable to those found previously with facial caricatures. The set of caricatured vocalisations provided open a promising line of research for investigating vocal affect perception and emotion processing deficits in clinical populations.

## 1 Introduction

We are highly skilled at recognising socially-relevant information in voices, from the age and gender of a speaker (Hartman & Danhauer, 1976; Lee, Byatt, & Rhodes, 2000; Mullennix, Johnson, Topcu-Durgun, & Farnsworth, 1995), to their emotional state (Elfenbein & Ambady, 2002). This latter ability – interpreting and identifying the emotions of others – is a crucial aspect of socially adaptive behaviour, allowing us to respond appropriately in social situations and to distinguish friend from foe. We can express emotions not only through the words we speak, but also through nonverbal vocalisations such as screams, laughs, and cries which closely parallel vocalisations in other species (Belin, Fecteau, & Bedard, 2004). These affective vocalisations can convey strong emotional content, and as such, provide a valuable tool not only for examining emotion recognition across cultures and species, but also impaired emotion processing in clinical populations.

Previous research on nonverbal vocalisations has revealed highly accurate recognition across a wide range of positive and negative emotions (Belin, Fillion-Bilodeau, & Gosselin, 2008; Lima, Castro, & Scott, 2013; Sauter & Scott, 2007; Schröder, 2003; Simon-Thomas, Keltner, Sauter, Sinicropi-Yao, & Abramson, 2009). In addition, affective vocalisations are recognised cross-culturally (Koeda et al., 2013; Laukka et al., 2013; Sauter, Eisner, Ekman, & Scott, 2010), and pre-linguistic infants are sensitive to their emotional content (Blasi et al., 2011). Nonverbal affective vocalisations provide an important bridge to emotion recognition in faces, where there is similarly no accompanying verbal information (Scott, Sauter, & McGettigan, 2010). Emotion processing in faces has been more extensively researched compared to voices, and research using affective vocalisations can provide key evidence for common mechanisms across voice and face perception (Belin et al., 2004; Belin, Bestelmeyer, Latinus, & Watson, 2011). In the present study we aim to examine the perception of caricatured emotions – a novel set of affective vocalisations – to investigate if emotion perception is modulated by caricaturing, akin to previous work with faces (Benson & Perrett, 1991; Calder et al., 2000; Calder, Young, Rowland, & Perrett, 1997; Lee et al., 2000; Rhodes, Brennan, & Carey, 1987). This is the first study to our knowledge that investigates recognition of vocal caricatures, and therefore provides a valuable addition to the field in understanding how vocal emotion is recognised and represented.

One central strand of research incorporating both facial and vocal emotion processing has been the investigation of emotional morphs, in which a continuum is created between two faces or voices expressing different emotions. The resulting stimuli are natural, well-controlled blends of a given pair of emotions (e.g. 25% fear/75% disgust). Studies using emotion morphs have been influential in demonstrating categorical perception of emotion in faces (Beale & Keil, 1995; Calder, Young, Perrett, Etcoff, & Rowland, 1996; Etcoff & Magee, 1992; Young et al., 1997) and in voices (Laukka, 2005), showing that linear changes in the stimuli result in non-linear shifts in emotion perception at the category boundary. In addition to creating morphs between emotions, we can extrapolate beyond the original emotions to generate a caricature emotion. Caricatured facial expressions, which exaggerate the facial features that differ between a given emotion and, for example, a neutral expression (such as the size of the eyes), are recognised more quickly than the original face (Calder et al., 1997; Calder et al., 2000; Rhodes et al., 1987) and are rated as more distinctive than the original (Lee et al., 2000). At the same time, anti-caricatures, which reduce the distinctiveness of the relevant features, result in slower recognition compared to the original face (Calder et al., 1997; Calder et al., 2000). Emotion intensity ratings for caricatures furthermore show a linear increase as the caricature intensity increases, suggesting that caricatures enhance emotional intensity (Benson, Campbell, Harris, Frank, & Tovée, 1999; Calder et al., 2000). Neuroimaging evidence has also demonstrated that regions involved in processing of fear and disgust are sensitive to the level of face caricature (Phillips et al., 1997). Caricature research can therefore play a key role in informing theories of how emotions and distinctiveness are represented and perceived, where results from different modalities and different stimulus manipulations should be taken into account.

Facial caricatures have played an influential role in the debate between prototype-based and exemplar-based theories of multi-dimensional emotion representation (Byatt & Rhodes, 1998; Calder et al., 2000; Valentine, 1991; Valentine & Bruce, 1986; Valentine, Lewis, & Hills, 2016). The prototype-based or norm-based model hypothesises that expressions are coded relative to a central representation in multi-dimensional space, with the distinctiveness of the expression determined by the length of the vector from the norm, and the identity determined by the direction of the vector. Thus, caricaturing would increase the distance from the norm (increasing the magnitude of the vector while the direction remains the same), resulting in improved recognition (Valentine et al., 2016). In the exemplar-based or absolute coding model (Valentine, 1991), all exemplars are represented without reference to a central norm or prototype, and the distance between expressions determines the similarity between them. One hypothesis is that caricatured expressions could sit in areas of low density in terms of other stored exemplars, making them more distinctive and therefore easier to recognise (Lee et al., 2000). Thus, both models can predict the caricature effects previously reported, and the issue of which model can more accurately explain the effects of caricaturing remains unresolved (Lewis, 2004; Valentine et al., 2016).

The use of a paired similarity rating task provides a valuable test for prototype- and exemplar-based models of vocal emotion by extracting two measures: firstly, we can assess the density of each stimulus (its similarity to all other stimuli) using the mean similarity to the full stimulus set; secondly, we can assess the similarity of each stimulus to the most average stimulus, providing a measure of where each stimulus sits relative to the prototype or norm. The key test will be to evaluate which model better predicts recognition as defined by reaction times in the emotion categorisation task.

**Fig. 1:**
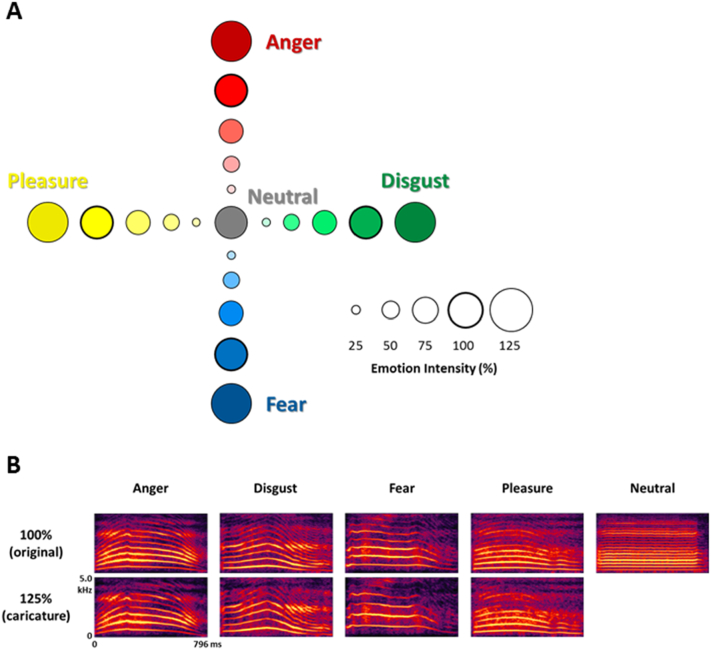
A) Stimulus emotion space (anger, disgust, fear, pleasure, and neutral) with each morphing level (25% steps) indicated by a circle. Original emotions (100%) with no morphing are indicated by a thicker circle. B) Spectrographs of female speaker for five original emotions (100%) and 125% caricatures.

The present study includes a set of morphs generated between nonverbal vocalisations expressing four emotions (anger, disgust, fear, and pleasure) and a neutral emotion, at 25% steps between each emotion. In addition, a caricature morph was created between the neutral emotion and each of the four emotions, which was extrapolated 25% beyond the original emotion (i.e. 125% anger, disgust, fear, and pleasure with neutral as the reference; see Fig. 1). We validated the set of morphs and caricatures using several behavioural measures: emotion intensity ratings on the four emotional scales, valence and arousal ratings, speeded forced-choice emotion categorisation and paired similarity ratings. Thus, we incorporate both categorical measures and dimensional measures (arousal and valence) of vocal emotion processing, providing a broader characterisation of the perception of affective vocalisations.

Based on previous research using facial caricatures, we would hypothesise: a monotonic increase in emotion intensity ratings (specific to that emotion), faster reaction times in categorising each emotion, and increased arousal ratings as the caricature intensity increases. Previous studies have not examined dimensional ratings in detail; in line with intensity ratings, we would hypothesise increased arousal ratings and more extreme valence ratings (more negative for negative emotions; more positive for positive emotions) as the caricature intensity increases. Conversely, we would hypothesise slower reaction times for anti-caricatures (25%, 50%, 75%) compared to the original emotion, as well as lower emotion intensity ratings and lower arousal ratings, consistent with previous facial caricature studies.

Similarity ratings and emotion categorisation RTs will be used to evaluate prototype-based and exemplar-based accounts of emotion recognition. Based on previous studies using facial caricatures (Johnston, Milne, Williams, & Hosie, 1997; Lee et al., 2000; Valentine, 1991), we would predict that the 125% caricatures will be less densely represented (sit in areas of lower density) and sit farther from the prototype stimulus compared to anti-caricatures and ambiguous morphs (e.g. 50% fear/50% anger). However, it is less clear if caricature effects can be better reflected by a prototype-based or a norm-based account (Valentine et al., 2016). We focus on assessing the unique contribution of the two accounts in the recognition of affective vocalisations.

## 2 Methods

### 2.1 Stimuli

Stimuli consisted of nonverbal affective vocalisations from the Montreal Affective Voices database (Belin et al., 2008) produced by two actors (one male, one female). Each actor produced five emotional expressions using the vowel /a/: anger, disgust, fear, pleasure and neutral. From these five vocalisations, stimuli were generated by morphing between each vocalisation and the neutral stimulus from the same speaker (see Fig. 1).

Voice morphing was performed using STRAIGHT (Kawahara & Matsui, 2003) in Matlab (Mathworks, Inc., Natick, USA). STRAIGHT performs an instantaneous pitch-adaptive spectral smoothing in each stimulus for separation of contributions to the voice signal arising from the glottal source versus supralaryngeal filtering. STRAIGHT allows modifying voice stimuli along five dimensions – f0, frequency, duration, spectrotemporal density, and aperiodicity – that can be manipulated and combined across stimuli independently of one another. Time-frequency landmarks to be put in correspondence across voices during morphing were manually identified in each stimulus, and corresponded to the frequencies of the first three formants at onset and offset of phonation. Morphed stimuli were then generated by resynthesis based on the linear (time and aperiodicity) and logarithmic (f0, the frequency structure and spectrotemporal density) interpolation of these time–frequency landmarks. Stimuli were normalized in energy (root mean square) before and after morphing.

Two morphing continua were produced: 1) between neutral and each of the four emotions (neutral-anger, neutral-disgust, neutral-fear, and neutral-pleasure), and 2) between pairs of emotions (anger-disgust, anger-fear, anger-pleasure, disgust-fear, disgust-pleasure, and fear-pleasure). The first morphing continuum, between neutral and each emotion, consisted of 6 stimuli progressing in acoustically-equal steps of 25% - e.g. neutral (N) to anger (A): 100% N; 75% N/25% A; 50% N/50% A; 25% N/75% A; 100% A; 125% A. The 125% emotion was generated by extrapolating along the neutral-emotion dimension to create a caricatured vocalisation (see Fig. 1). The second morphing continuum, between pairs of emotions, consisted of 5 stimuli progressing in acoustically-equal steps of 25% - e.g. anger (A) to fear (F): 100% A; 75% A/25% F; 50% A/50% F; 25% A/75% F; 100% F. Morph stimuli of this kind have been previously used to investigate the perception and neural representation of vocal affect (Bestelmeyer, Kotz, & Belin, 2017; Salvia et al., 2014). Stimulus duration was normalized to the average duration of 796 ms using pitch-preserving time-stretching algorithms using commercially available algorithms (iZotope Radius™), low-pass filtered at 5 kHz and finally normalized in root mean square amplitude. In total, 78 stimuli were used in the experiment, consisting of 39 stimuli for each speaker.

### 2.2 Procedure

Participants performed three behavioural tasks across three subsequent different-day sessions: paired similarity ratings (sessions 1 and 2), and categorical and dimensional ratings as well as speeded emotion categorisation (session 3). Categorical/dimensional ratings and categorisation were only performed during the last session to avoid biases towards either categorical or dimensional features during the similarity ratings. In the similarity ratings task, participants rated the perceived similarity of all (within-speaker) pairs of stimuli in the absence of instructions about any particular stimulus feature that would drive their ratings. On each of two sessions, participants rated the similarity between all stimuli from a given speaker (speaker order counterbalanced across participants). On each trial, they were presented with one of the possible 741 pairs of sounds, and were asked to rate how similar they were on a scale of “very similar” to “very dissimilar.” They could listen to the pair of stimuli as many times as necessary before giving a response. Participants were given 10 practice trials using a set of 10 vocalisations that were not included in the main experiment. The total duration of each session was approximately 120 min.

Participants performed the emotional ratings and speeded categorisation tasks in alternating blocks in the same (final) session. In the ratings task, participants rated each stimulus on arousal (low to high), valence (negative to positive), and emotional intensity for four emotions (anger/disgust/fear/pleasure, low to high). In all ratings tasks, data were coded on a scale of 0 (low) to 1 (high). In the categorisation task, participants were instructed to identify as quickly as possible the emotion expressed for each stimulus as being anger, disgust, fear, or pleasure. The association between a particular emotion and a particular response button was randomised for each block. Before the session began, participants were given 10 practice trials for both the rating and categorisation task. Participants were familiarised to the entire stimulus set before the first block for each speaker.

The emotion ratings and categorisation session consisted of 12 blocks, incorporating 6 blocks of each task (rating and categorisation). Each task was repeated three times for each of the two speakers (1 male, 1 female), and data were averaged across the three repetitions. One block contained all 39 stimuli for one speaker, and stimulus order was randomised for each participant. Speaker gender order and task order were pseudo-randomised for each participant, such that each gender and each task could only appear a maximum of twice in a row. The total duration of the session was approximately 120 min.

### 2.3 Statistical analysis

For the caricature analysis, we assessed the relationship between morphing level and the behavioural measures with a linear mixed-effects analysis as implemented in the lme4 R package (Bates, Maechler, Bolker, & Walker, 2014). Ratings and reaction times were included as dependent variables (in separate analyses), with morphing level (25% to 125%), speaker gender, and the interaction between morphing level and speaker gender as predictors. Subjects were modelled as random effects, with by-subject random intercepts and slopes for morphing level. The Satterthwaite approximation for degrees of freedom (Satterthwaite, 1946) was used as implemented in the lmerTest R package (Kuznetsova, Brockhoff, & Christensen, 2015). In the categorisation task, RTs above 2.5 s were discarded (5.8% of total responses).

For the similarity ratings analysis, comparing prototype- and exemplar-based accounts of emotion recognition, we considered data from the similarity ratings and speeded emotion categorisation tasks. For each speaker and participant, we extracted the similarity rating between each pair of stimuli – coded between 0 (“very similar”) and 1 (“very dissimilar”) – and the mean reaction time for each stimulus (averaged across the three repetitions) from the emotion categorisation task. From the similarity ratings we defined two measures: stimulus (exemplar) density, a measure of how similar each stimulus is to the entire stimulus set; and similarity to the average, a measure of the distance to the prototypical or average voice stimulus. For the exemplar density measure, we extracted the mean similarity value for each stimulus compared to all other stimuli, with 0 indicating higher similarity to other stimuli (and higher density). For the similarity to average measure, we defined the most average stimulus as the stimulus with the highest mean similarity (defined separately for each speaker). For each participant, we extracted the similarity rating for each stimulus compared to the average stimulus. First, we assessed the similarity between caricatures and the original (100%) emotions with respect to the two measures, given previous evidence showing that caricatures sit in areas of lower density and are further from the central stimulus (Johnston et al., 1997; Lee et al., 2000). Secondly, using semi-partial correlation we investigated the relationship between reaction time and the two measures (stimulus density and similarity to average) to assess the unique contribution of each measure to vocal emotion recognition time.

### 2.4 Participants

Ten volunteers took part in the study (5 female; mean age: 25.1). All participants were right-handed, and were tested for normal hearing. They provided informed written consent and received monetary compensation for their participation. The local ethics committee (College of Science and Engineering, University of Glasgow) approved the study.

## 3 Results

### 3.1 Perception of caricatures

#### 3.1.1 Arousal and valence

**Fig. 2:**
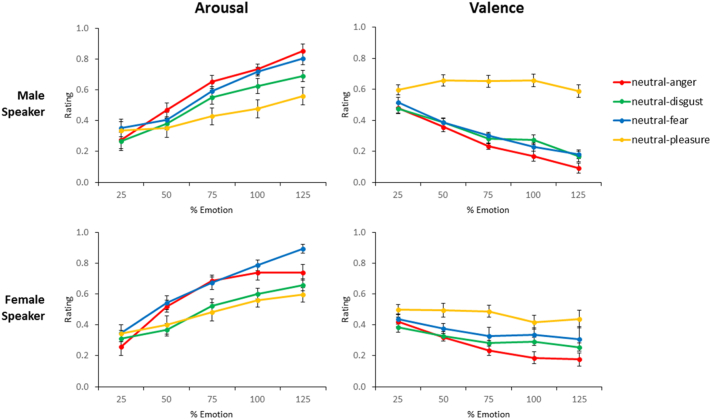
Mean ± SE for arousal (left) and valence (right) ratings for anger, disgust, fear, and pleasure stimuli across morphing levels (25% to 125%). Ratings are coded on a scale of 0 (low) to 1 (high) for arousal, and 0 (negative) to 1 (positive) for valence.

Linear mixed-effects analyses revealed a strong effect of morphing level on arousal and valence ratings (see Table 1 for full results). There was a significant increase in arousal ratings for all four emotions as morphing level increased towards 125%, with no significant effect of speaker gender or an interaction between morphing level and gender. Morphing level also modulated valence ratings, albeit only for negative emotions. Anger and disgust showed a significant decrease in valence ratings as morphing level increased from 25% to 125% (see Table 1 and Fig. 2). Fear stimuli showed a significant interaction between morphing level and speaker gender, with post-hoc tests revealing a significant decrease in valence ratings for the male speaker only (male: β = −0.33, SE = 0.04, t(9) = −8.38, p < .0001; female: β = −0.12, SE = 0.10, t(9) = −1.14, p > .05). Anger and disgust also showed a significant interaction between morphing level and speaker gender, with both speakers showing a significant decrease in valence ratings. A stronger morphing effect emerged for the male speaker for anger stimuli (male: β = −0.39, SE = 0.05, t(9) = −8.57, p < .0001; female: β = −0.25, SE = 0.05, t(9) = −5.37, p < .001) as well as for disgust stimuli (male: β = −0.29, SE = 0.04, t(9) = −7.40, p < .0001; female: β = −0.16, SE = 0.04, t(9) = −2.85, p < .05).

**Table 1:** Linear mixed effects analysis of arousal and valence ratings across all morphing levels (25%–125%), showing beta coefficients, standard errors and t values for ratings of the four emotions (anger, disgust, fear, pleasure). * indicates p < .05; ** indicates p < .01; *** indicates p < .001.

Stimulus Emotion	Fixed Effects	Arousal			Valence		
		β	SE	t	β	SE	t
**Anger**	Morph	0.47	0.07	6.59***	-0.25	0.05	-5.47***
	Speaker	0.06	0.05	1.27	0.10	0.03	3.35**
	Morph x Speaker	0.09	0.06	1.58	-0.14	0.04	-3.78***
**Disgust**	Morph	0.37	0.07	5.46***	-0.12	0.04	-2.97**
	Speaker	0.04	0.05	<1	0.14	0.03	4.40***
	Morph x Speaker	0.07	0.06	1.13	-0.18	0.04	-4.53***
**Fear**	Morph	0.54	0.06	8.46***	-0.12	0.07	-1.83
	Speaker	0.04	0.04	<1	0.12	0.06	1.98
	Morph x Speaker	0.05	0.05	1.04	-0.21	0.08	-2.80**
**Pleasure**	Morph	0.26	0.05	5.01***	-0.08	0.05	-1.67
	Speaker	0.02	0.06	<1	0.11	0.06	1.87
	Morph x Speaker	0.03	0.07	<1	0.08	0.07	1.10

#### 3.1.2 Emotion intensity ratings

**Fig. 3:**
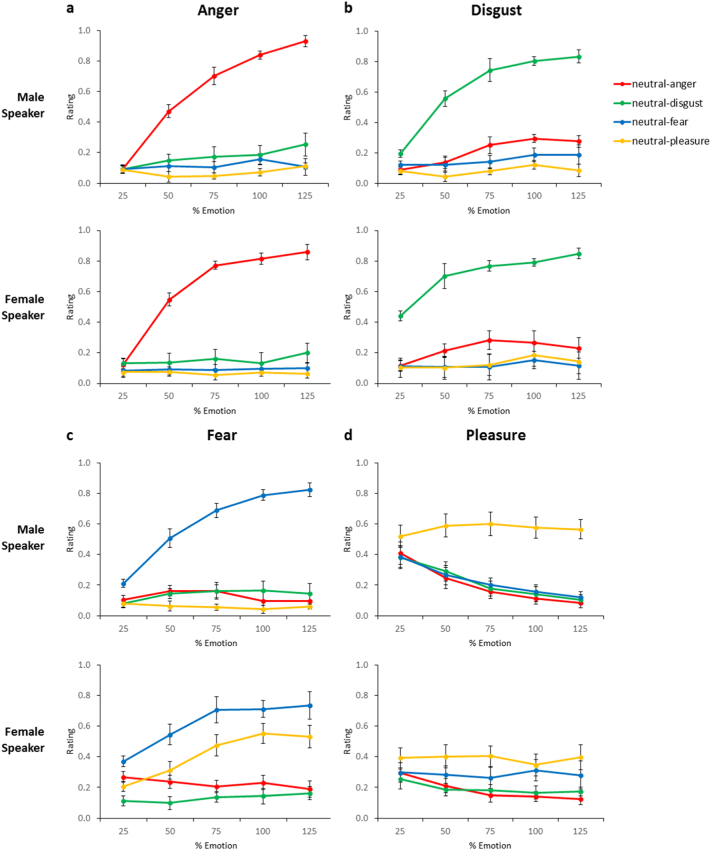
Mean ± SE for emotion intensity ratings across morphing levels: (a) anger, (b) disgust, (c) fear, and (d) pleasure ratings. Ratings are coded on a scale of 0 (low) to 1 (high).

The linear mixed-effects analysis was conducted on within-emotion ratings (e.g. anger ratings for anger stimuli, fear ratings for fear stimuli, etc.; see Table 2 for a complete breakdown of the results). For anger ratings, the results indicated that morphing level was a strong predictor for anger stimuli with no effect of speaker gender. There was a significant increase in anger intensity ratings for anger stimuli as morphing level increased from 25% to 125% (see also Table 3 and Fig. 3). A similar pattern was found for disgust and fear ratings, along with an additional significant interaction between morphing level and speaker gender, with post-hoc tests revealing a significant increase in disgust intensity ratings for both speakers (male: β = 0.61, SE = 0.06, t(9) = 9.96, p < .0001; female: β = 0.36, SE = 0.07, t(9) = 4.86, p < .001) and a significant increase in fear intensity ratings for both speakers (male: β = 0.60, SE = 0.07, t(9) = 8.73, p < .0001; female: β = 0.36, SE = 0.12, t(9) = 3.01, p < .05). For pleasure intensity ratings, the morphing level did not show a significant effect on pleasure stimuli. Thus, for the three negative emotions, there was a strong linear increase in emotion intensity ratings as morphing level increased, in line with previous facial caricature studies (Benson et al., 1999; Calder et al., 2000). Both dimensional features showed a strong linear relationship between morphing and ratings, consistent with our hypotheses about the role of caricaturing in enhancing both dimensional and categorical intensity ratings.

**Table 2:** Linear mixed effects analysis of emotion intensity ratings across all morphing levels for within-emotion ratings (e.g. anger intensity for anger stimuli, disgust intensity for disgust ratings, etc.). * indicates p < .05; ** indicates p < .01; *** indicates p < .001.

Stimulus Emotion	Fixed Effects	Rating of Emotion Intensity		
		β	SE	t
**Anger**	Morph	0.69	0.07	10.28***
	Speaker	0.11	0.07	1.63
	Morph x Speaker	0.12	0.08	1.52
**Disgust**	Morph	0.36	0.07	5.31***
	Speaker	0.27	0.06	4.37***
	Morph x Speaker	0.25	0.07	3.32**
**Fear**	Morph	0.36	0.09	3.96***
	Speaker	0.19	0.09	2.14*
	Morph x Speaker	0.24	0.11	2.26*
**Pleasure**	Morph	-0.02	0.06	<1
	Speaker	0.14	0.06	2.21*
	Morph x Speaker	0.05	0.08	<1

**Table 3:**
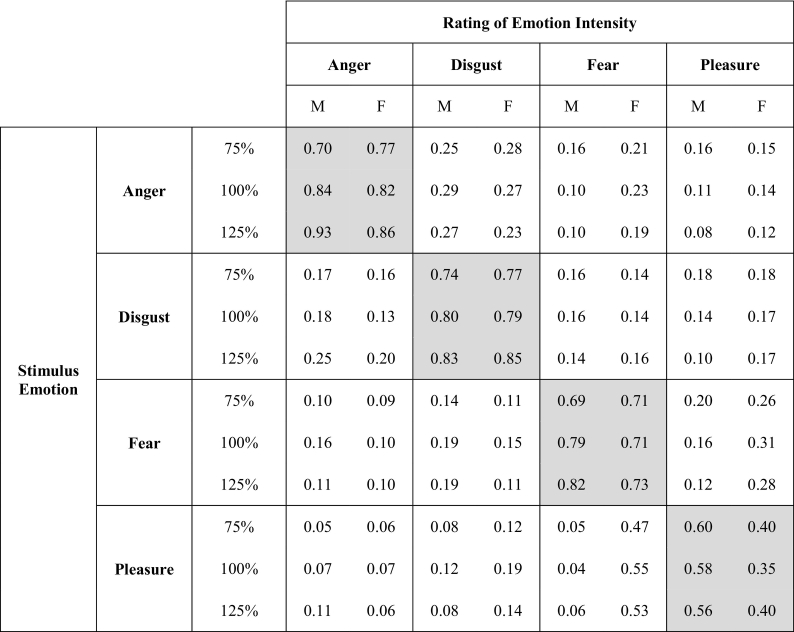
Mean emotion intensity ratings at 75%, 100%, and 125% morphing levels, on a scale of 0 (low) to 1 (high). Shaded areas indicate within-emotion ratings (e.g. anger ratings for anger stimuli).

#### 3.1.3 Emotion categorisation

**Fig. 4:**
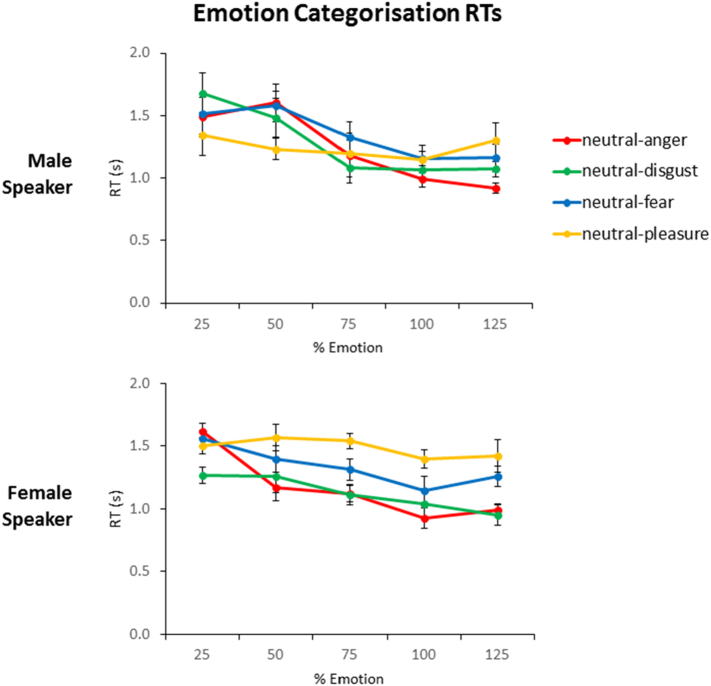
Mean ± SE for speeded emotion categorisation reaction times across morphing levels.

The linear mixed-effects analysis for the speeded emotion categorisation task was conducted on RTs for the four emotions separately. The results indicated that morphing level was a strong predictor of the variance for the anger, disgust, and fear stimuli (results are summarised in Table 4), showing a significant decrease in RTs for the three negative emotions as morphing level increased from 25% to 125% (see also Fig. 4). Disgust showed a further significant interaction between morphing level and speaker gender, with post-hoc tests revealing a significant effect of morphing level for both speakers (male: β = −0.65, SE = 0.12, t(9) = −5.51, p < .0001; female: β = −0.36, SE = 0.08, t(9) = −4.54, p < .001). This supports the evidence from facial caricature studies showing that categorisation becomes faster as the emotion becomes more distinctive (Calder et al., 1997; Rhodes et al., 1987).

**Table 4:** Linear mixed effects analysis of speeded emotion categorisation reaction times across all morphing levels for RTs of the four emotions (anger, disgust, fear, pleasure). * indicates p < .05; ** indicates p < .01; *** indicates p < .001.

Stimulus Emotion	Fixed Effects	Reaction Time		
		β	SE	t
**Anger**	Morph	-0.60	0.12	-5.10***
	Speaker	0.15	0.11	1.34
	Morph x Speaker	-0.10	0.14	<1
**Disgust**	Morph	-0.35	0.09	-3.82***
	Speaker	0.38	0.10	3.64***
	Morph x Speaker	-0.30	0.12	-2.44*
**Fear**	Morph	-0.34	0.13	-2.68*
	Speaker	0.11	0.12	<1
	Morph x Speaker	-0.12	0.14	<1
**Pleasure**	Morph	-0.13	0.11	-1.21
	Speaker	0.29	0.12	2.40*
	Morph x Speaker	0.06	0.14	<1

### 3.2 Prototype vs. exemplar density

To determine exemplar density, we computed the mean similarity rating for each stimulus compared to all stimuli (N = 39) for each speaker, with ratings coded between 0 (“very similar”) and 1 (“very dissimilar”). A stimulus with a low rating would be more similar to the stimulus set and thus sit in an area of higher density. The most average stimulus was 75% pleasure/25% fear for the male speaker (M = 0.42, SE = 0.028) and 75% pleasure/25% anger for the female speaker (M = 0.41, SE = 0.032). In contrast, the most dissimilar stimulus for each speaker was a 125% caricature: 125% anger for the male speaker (M = 0.67, SE = 0.035) and 125% fear for the female speaker (M = 0.68, SE = 0.038). To determine similarity to the prototype, we computed the similarity rating of each stimulus to the most average stimulus for each speaker (M = 75% pleasure/25% fear; F = 75% pleasure/25% anger). Therefore, for each stimulus we have a measure of its average density (i.e. average similarity rating) and its similarity to the prototype.

Firstly, we assessed if there was a systematic change in density or distance from prototype for the caricatures compared to the veridical emotions, as shown previously in facial caricatures (Lee et al., 2000). In a linear mixed-effects analysis, average density and similarity to prototype were included as dependent variables (in separate analyses), with morphing level (100% and 125%), speaker gender, and the interaction between morphing level and speaker gender as predictors. Subjects were modelled as random effects. As above, the Satterthwaite approximation for degrees of freedom (Satterthwaite, 1946) was used as implemented in the lmerTest R package (Kuznetsova et al., 2015).

In the average density analysis, there was a significant main effect of morphing level (*F*(1, 147) = 26.01, p < .0001) but no effect of speaker gender (*F*(1, 147) = 3.59, p > .05) and no interaction between morphing level and speaker gender (*F* < 1). Pairwise comparisons showed a significant difference between the original and caricature morphs (p < .0001), with caricatures rated as less similar to the stimulus set (i.e. lower density). In the similarity to prototype analysis, there was also a significant main effect of morphing level (*F*(1, 147) = 4.50, p < .05) and no effect of speaker gender (*F*(1, 147) = 1.45, p > .05) or interaction between morphing level and speaker gender (*F* < 1). Pairwise comparisons again showed a significant difference between the original and caricature morphs (p < .05), with caricatures rated as less similar to the average stimulus.

We then assessed the unique contribution of each measure in emotion recognition as measured by the semi-partial correlation with reaction times in the speeded emotion categorisation task using. For each participant, we computed the semi-partial correlation for all stimuli (independently for the two speakers) between reaction time and each measure. Similarity to prototype was a significant predictor of reaction times, independently of average density (*t*(9) = −3.49, p < .01; M = −0.10, SE = 0.029); thus, as distance from prototype increases, reaction time decreases. In contrast, average density was not a significant predictor of reaction time, independently of similarity to prototype (*t*(9) = −2.01, p = .08; M = −0.07, SE = 0.035).

## 4 Discussion

The aim of the present study was to assess the perception of caricatured and morphed emotions in nonverbal vocalisations across a range of behavioural tasks. This would provide the first test of whether caricature effects in voices mirror findings in the face literature. Furthermore, previous studies have not focussed on dimensional ratings – arousal and valence – which we have incorporated along with emotion intensity ratings and speeded emotion categorisation. We include a further investigation of the unique contribution of similarity to prototype and average density to emotion recognition, two key measures that have been previously examined with reference to caricatures (Valentine et al., 2016).

Results from the rating tasks (emotion intensity, arousal, and valence) revealed a significant linear increase in emotion intensity ratings as morphing level increased towards the 125% emotion – in line with results from facial caricatures (Calder et al., 2000; Rhodes et al., 1987) – as well as an increase in arousal ratings and a decrease in valence ratings. Anti-caricatures (25%, 50% and 75%) showed lower intensity and arousal ratings, while the caricatures (125%) showed higher ratings compared to the original emotions. The caricature and anti-caricature effects were robust and generalised across the two speakers. The linear increase in arousal ratings applied to all four emotions; however, for emotion intensity ratings only the negative emotions showed a significant increase. Valence ratings showed a linear decrease as morphing level increased for negative emotions, indicating that the caricature emotions were perceived as not only more intense, but also more negative.

The speeded emotion categorisation task revealed a consistent pattern across the three negative emotions, with reactions times decreasing linearly as the morphing level increased from 25% towards the caricature emotion (125%). This is consistent with research on facial caricatures (Calder et al., 1997; Calder et al., 2000; Lee et al., 2000), suggesting that caricaturing facilitated emotion recognition and generated a more distinctive emotion. However, the positive emotion (pleasure) did not show a significant shift in reaction times based on the morphing level, showing a similar pattern to the valence ratings. In general, we found a high confusability between fear and pleasure for the female speaker (see Fig. 2C/D), indicating that the slow reaction times may be due to similarity in the two emotions as the morphing level increased towards the caricature.

Recognition rates for the pleasure emotion in previous affective vocalisation studies were at 62% (Belin et al., 2008) and 61.6% (Sauter & Scott, 2007), whereas higher recognition rates above 80% have been reported for other positive emotions such as happiness (Belin et al., 2008) and achievement (Sauter & Scott, 2007). Though we have only one positive emotion in the present study, previous findings would indicate that the inconsistent results for pleasure are not due to a feature of positive emotions more generally. The lack of caricaturing effect and low recognition rates suggest that the original female pleasure stimulus was not ideally suited for eliciting that emotion. Previous research has demonstrated that pleasure vocalisations can be confused with emotions such as contentment (Sauter, Eisner, Calder, & Scott, 2010), and that actors struggled to produce a sensual pleasure vocalisation stimulus (Sauter & Scott, 2007). It is also possible that the anomalous effects for female pleasure could be explained in the present study by the presence of a confusable alternative emotion (fear; see Fig. 3C/D): we found that arousal ratings showed an increase as caricaturing increased – suggesting that caricaturing does generate a more intense emotion – while at the same time, ratings of fear intensity increased for the female pleasure stimuli. Further testing would be necessary in order to obtain a more accurate pleasure stimuli. There has been a predominance of negative emotions used throughout many studies, though recent studies have established a larger set of positive emotions such as amusement and relief (Sauter, 2010; Sauter & Scott, 2007). This emphasises further the need to incorporate additional positive emotions in future studies in order to assess the caricature effect in more detail.

The analysis of similarity ratings revealed evidence that similarity to prototype plays a unique role in emotion reaction, in contrast to exemplar density. We did not find sufficient evidence to confirm the exemplar density model. In the contrast of original (100%) and caricature (125%) emotions, we found that caricatures are both less similar to the prototype and less similar to all other stimuli (i.e. in an area of lower density), consistent with previous evidence using caricatured faces (Valentine et al., 2016). The semi-correlation results furthermore point to a unique role for the prototype-based model which cannot be explained by the exemplar density model. Previous studies have employed multi-dimensional scaling to address the representation space of emotion recognition (Lee et al., 2000), and our results using raw similarity ratings and reaction times support these findings.

Our results are consistent with a multi-dimensional prototype model, in which distinctiveness through caricaturing can enhance both emotion intensity and ease of recognition. The linear increase in emotion intensity and dimensional ratings, as well as the linear decrease in reaction times, as morphing increased from 25% to 125% can be explained by the increasing distance from the central prototype. The paired similarity ratings provide further evidence that similarity to the prototype contributes to emotion recognition independently of exemplar density. Converging evidence for norm-based or prototype coding has come from studies of voice recognition (Andics et al., 2010; Latinus & Belin, 2011; Latinus, McAleer, Bestelmeyer, & Belin, 2013; Mullennix et al., 1995; Papcun, Kreiman, & Davis, 1989), face recognition (Leopold, O'Toole, Vetter, & Blanz, 2001; Loffler, Yourganov, Wilkinson, & Wilson, 2005), and facial expression recognition (Skinner & Benton, 2010). For instance, Latinus and Belin (2011) demonstrated that the strongest adaptation effects for a vocal stimulus was the matched “anti-voice” which lies opposite a central, average voice. Further studies could investigate vocal caricatures within an adaptation paradigm to assess these stimuli more directly in the context of a prototype account.

The convergence of results from face and voice research with respect to processing of morphs and caricatures points to analogous processing mechanisms in the two modalities (Belin et al., 2011). As in previous facial caricature studies (Calder et al., 1997; Calder et al., 2000; Young et al., 1997), we report that caricaturing of affective vocalisations results in enhanced perception of emotion intensity and arousal as well as faster reaction times in emotion categorisation. Although the present study did not directly examine perception across both voices and faces, these parallel results point to the presence of multimodal interactions. The ‘auditory face’ model of voice perception (Belin et al., 2004) argues for a set of independent processing stages for the analysis of speech information, affective information and vocal identity, along with interactions between voice and face processing at each stage. Faces and voices carry similar socially-relevant information, such as identity and emotional state; as such, it would be beneficial to combine affective information into a more complete, supramodal emotional percept (Schirmer & Adolphs, 2017).

There are several additional issues that could be addressed in future research to extend the research on vocal caricatures. Firstly, the level of caricaturing in the present study was set at 125%, which produced a naturalistic vocalisation. However, it is unclear how further exaggeration would modulate emotion recognition. Previous results from facial caricatures revealed that emotion intensity ratings continued to increase for caricatures up to 175%, despite the fact that naturalness ratings decreased (Calder et al., 2000). It would therefore be of interest to investigate if emotion effects in vocal caricatures extend beyond naturalness limits at levels of caricature of 150% and above. Secondly, we have used two speakers to investigate possible generalisations across speakers (one male, one female), but a larger set of speakers of both genders would be informative in examining possible voice gender effects in emotion processing. Finally, we have made use of brief, nonverbal expressions to study emotion processing in the absence of linguistic cues, akin to the face domain. However, further research using longer, more naturalistic speech is necessary in order to understand how our results generalise to different types of utterances.

These results have important implications for interventions aimed at enhancing emotion recognition and emotion processing deficits in clinical populations. Previous research has demonstrated that recognition is more accurate when faces are learned as caricatures compared to the original (100%) faces (Itz, Schweinberger, & Kaufmann, 2017; Kaufmann & Schweinberger, 2012). This learning advantage for caricatures is generalizable – improving recognition of the original face when it was learned as a caricature (Itz et al., 2017) – suggesting that caricatures could act as an aid for those with poor emotion recognition in both visual and auditory domains. Frontotemporal dementia patients showed improved emotion recognition for facial caricatures compared to the original emotions (Kumfor et al., 2011), and extensive research has shown impaired vocal affect processing in schizophrenia (Hoekert, Kahn, Pijnenborg, & Aleman, 2007; Kucharska-Pietura, David, Masiak, & Phillips, 2005; Leitman et al., 2005), depression (Naranjo et al., 2011) and Parkinson's disease (Breitenstein, Van Lancker, Daum, & Waters, 2001; Dara, Monetta, & Pell, 2008). Our results suggest that vocal caricatures can generate emotions that are more intense and distinctive, and the present set of caricatured vocalisations could act as a valuable rehabilitation tool for enhancing emotion processing in the auditory domain.

To conclude, we have generated a set of homogeneous affective vocalisations with wide variations in acoustical and emotional parameters, with naturalistic caricatures that are perceived as a better likeness than the veridical emotions. Our results demonstrate for the first time that caricaturing vocal expressions can enhance the intensity, arousal and distinctiveness of the emotion in a comparable way to previous work using facial caricatures, underscoring the parallels in the underlying mechanisms for facial and vocal emotion. This advantage for caricatures over the original emotion supports the notion that emotions are represented in terms of their deviation from the norm (Benson & Perrett, 1991; Rhodes et al., 1987), and provides a promising venue for investigating emotion impairments in clinical populations.

## CRediT authorship contribution statement

**Caroline M. Whiting:** Conceptualization, Methodology, Validation, Formal analysis, Investigation, Data curation, Writing - original draft, Writing - review & editing, Visualization. **Sonja A. Kotz:** Writing - original draft, Writing - review & editing, Funding acquisition. **Joachim Gross:** Methodology, Writing - original draft, Writing - review & editing, Supervision, Project administration, Funding acquisition. **Bruno L. Giordano:** Methodology, Software, Validation, Formal analysis, Investigation, Resources, Data curation, Writing - original draft, Writing - review & editing, Supervision, Funding acquisition. **Pascal Belin:** Conceptualization, Methodology, Software, Resources, Writing - original draft, Writing - review & editing, Supervision, Funding acquisition.
